# Vermiform Appendix and the Potential for Missed Pathologies

**DOI:** 10.7759/cureus.25055

**Published:** 2022-05-16

**Authors:** Bidish K Patel, Ashish R Singh, Sandyya Umamahesweran, Bhawana Ashok Badhe

**Affiliations:** 1 Pathology, Jawaharlal Institute of Postgraduate Medical Education & Research, Puducherry, IND; 2 Research, Massachusetts General Hospital Cancer Center, Boston, USA; 3 Pathology, Indira Gandhi Institute of Medical Sciences, Patna, IND

**Keywords:** appendix polyp, xanthogranulomatous appendicitis, appendix lymphoma, appendix carcinoid, appendix endometriosis, incidental findings in appendix, rare lesions in appendix, appendectomy, appendix histopathology, appendix tumors

## Abstract

Introduction

The appendix is considered an appendage of little value and is often treated disdainfully, be it as part of evolutionary process, on a grossing table, under a microscope or while archiving specimens and slides. It is only recently, with data indicating its importance in gut immunity and as the origin of pseudomyxoma, that its space in a human body appears vindicated.

Aim

Our aim was to screen the histopathologic spectrum of appendix lesions observed in our hospital for rare, incidental or clinico-radiologically uncertain lesions that would help emphasize a necessary seriousness in its sampling.

Method

All appendectomy specimens over ten years were screened for diagnosis other than acute/chronic/resolving appendicitis and pseudomyxoma peritonei. Among the recorded rare diagnoses, one representative case each, based on interesting history or pathology, was selected for discussion.

Observation

Forty-three lesions were found to meet inclusion criteria comprising 12 varied etiologies. Among these, 25 had a normal-appearing appendix and 27 were not suspected on radiology or on clinical/surgical assessment. Histopathology comprised, among others, neoplastic entities such as (Diffuse large B-cell) lymphoma, metastasis, carcinoid as well as interesting non-neoplastic diagnoses such as pinworm infestation (in the elderly) and (post-menopausal) endometriosis.

Conclusion

Sampling and histopathologic assessment of the appendix should be compulsory, careful and representative. Each specimen must be treated as harboring a potential pathology, until microscopically proven otherwise because missed “rare” diagnoses could delay therapy or alter key management decisions as cancer staging.

## Introduction

The appendix is considered an appendage of little value, its name probably derived from the synonym implying a postscript or supplement. Often condemned to the last pages of chapters, for surgeons and pathologists alike appendix embodies a troublesome organ notoriously prone to inflammation and perforation. However, once in a while, for those that care to dig, its little chamber of secrets does provide the odd surprise reminiscent of a jack-in-the-box toy.

Our article pertains to a series of some incidentally detected or unsuspected appendiceal lesions diagnosed over the past decade at our institute. While the lesions we will discuss are not very uncommon, the situations surrounding the diagnosis, for the most part, are. This bouquet of select cases we hope, serves as a reminder for the surgeons and pathologists to the multitude of lesions possible in the appendix. Hopefully, our cases also reinforce that dismissing the organ at the operating/grossing table may sometimes at least, be at the patients’ peril.

## Materials and methods

Our retrospective cross-sectional study encompasses cases that have been diagnosed in our institute over a period of about ten years (January 2007 - April 2017). Only microscopic re-evaluation of archival glass slides with tissue from vermiform appendix was performed. No new test was performed and patient anonymity was ensured. For such studies, institutional review board permission is waived off in our institute.

Old registers were searched for the keyword “Appendix” in the organ column and the corresponding entry in the diagnosis column was looked at. Cases of Perforation / Acute / Chronic appendicitis were excluded from further evaluation. Appendix findings in pseudomyxoma peritonei were also excluded since appendix involvement is inevitable either clinically and/or microscopically in such cases. The inclusion criteria comprised the other cases that were definitively diagnosed by the pathologist irrespective of the presence or absence of the lesion clinically, radiologically or while operating.

Subsequently, the clinical history of these cases was screened to search for interesting scenarios. Eventually, for each diagnosis, only one representative interesting case with unique history or workup findings was picked. The search was, thus, narrowed to 12 such scenarios. Demographic data and follow-up information, wherever available, were noted. The slides of these cases were retrieved from storage, the diagnoses reestablished and a representative image was captured.

The sampling of the appendix in our center depends on the gross appearance of the organ to the naked eye. For a normal-appearing appendix, one half of the tip (about 1 cm length) is sampled as a longitudinal section and another one random cross-section is taken from the body, usually near the resected base. Both these sections are processed in one block for microscopic evaluation. With obvious pathology, sampling is subjectively altered to address the lesion. Examples include more sections for primary appendiceal tumors including sampling of resected margin, sampling the entire appendix as serial cross-sections at 3-5 mm intervals for clinically suspected tumors and transverse section at the site of perforation.

## Results

The retrospective search yielded 43 cases pertaining to 12 different diagnoses. These include 12 cases (27.9%) of *Enterobius vermicularis*, i.e., pinworm infection, nine cases (20.9%) of appendiceal neuroma also known as fibrous obliteration, six cases (13.95%) of appendiceal carcinoid, four cases (9.3%) of mucocele, three cases (6.97%) of appendiceal tuberculosis, two cases each of xanthogranulomatous appendicitis and appendiceal lipoma (4.7% each), and one case each of appendiceal endometriosis, hyperplastic polyp, mucinous cystadenoma of the appendix, diffuse large B-cell lymphoma (DLBCL) and (micro)metastasis from an ovarian serous cystadenocarcinoma (2.3% each).

The histopathological findings and clinical context of one representative interesting case per diagnosis are discussed below.

**Figure 1:**
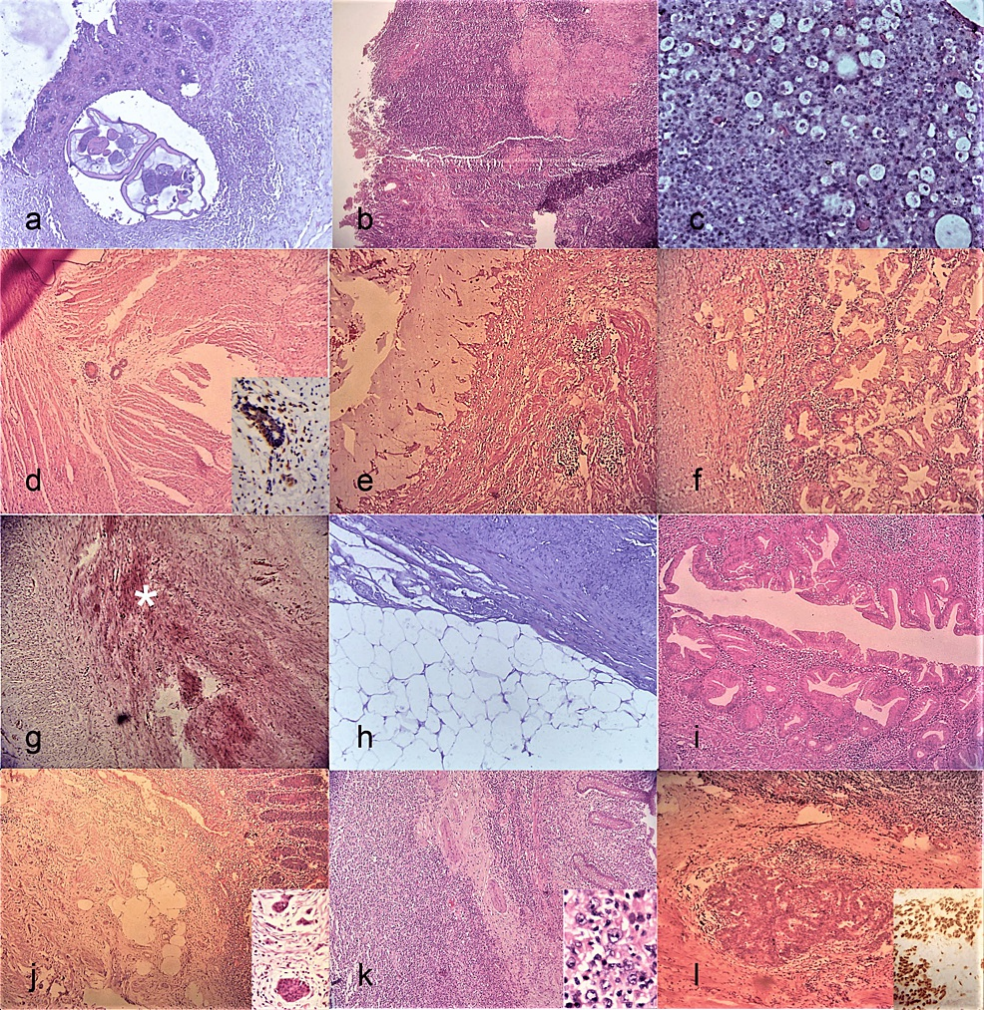
Rare lesions of the appendix. (a) Pinworm infection (H&E, 40X). (b) Granulomatous appendicitis (H&E, 40X). Coalescent granulomas and occasional Langhans giant cells seen. (c) Xanthogranulomatous appendicitis (H&E, 100X). The foamy macrophages stand out like a starry sky pattern. (d) Endometriosis (H&E, 40X). Entrapped within the muscularis propria, 3 glands are seen. Inset (IHC, 400X) shows positive immunohistochemistry (IHC) for Estrogen Receptor in the glands and surrounding endometrial stromal cells. (e) Mucocele (H&E, 40X). Abundant luminal mucin and attenuated mucosal lining. (f) Hyperplastic polyp (H&E, 100X). Note the classic serrated appearing and crowded glands. (g) Neuroma (H&E, 100X). (*) indicates the obliterated lumen with deep pink appearing nerve bundles surrounded on either side by the pale muscular walls of appendix. (h) Lipoma (H&E, 40X). Adipocytes seen abutting muscle bundles. (i) Mucinous cystadenoma (H&E, 100X). Dysplastic epithelium has replaced the normal lining. (j) Carcinoid (H&E, 40X). Left field shows the tumor and inset (H&E, 400X) shows the neoplastic cells in cords, islands and ribbons. (k) (Diffuse large B-cell) Lymphoma (H&E, 40X). Transmural involvement seen. Inset (H&E, 400X) shows a predominant large cell population. (l) Metastatic (Ovarian) cancer (H&E, 100X). Glandular pattern seen. Inset (IHC, 400X) shows IHC positivity for WT1 in these glands.

Case 1 was a 70-year-old man with proven retroperitoneal epithelioid sarcoma where appendicectomy was done to improve access for excising adequate margins. The appendix was normal externally. Histopathology showed part of a small nematode with chitinous wall and lateral spines lying freely in the lumen (Figure 1a). This was compatible with pinworm or *Enterobius vermicularis* infestation.

Case 2 represented a 25-year-old man with anorexia, nausea presenting with rebound tenderness in the right iliac fossa. His sampled appendix was normal on gross examination, but showed submucosal as well as serosal caseating epithelioid granulomas (Figure 1b). Ziehl-Neelsen stain did not reveal any acid-fast bacilli. No parasitic structures or fungal elements/foreign bodies were demonstrated in the granulomas/giant cells. A diagnosis of granulomatous appendicitis strongly suggestive of tuberculosis was rendered. Post-operative computerized tomography (CT) scan revealed necrotic intra-abdominal nodes which gave more credibility to the suggested etiology.

Case 3 was a 49-year-old female with suspected appendiceal malignancy on radiology. Gross examination showed thickened, bulky pale appendix with adherent fat which on histology showed dense inflammatory infiltrate rich in mature lymphocytes, plasma cells and a prominent population of foamy histiocytes with ingested debris (Figure 1c). However, the inflammation was not transmural, nor was there sufficient monotony in the lymphoid cells to warrant a suspicion of lymphoma. Eventually, a diagnosis of xanthogranulomatous appendicitis was rendered.

Case 4 was an elderly 81-year-old lady with an invasive cecal malignancy possibly developing from a previously diagnosed tubulovillous adenoma. The appendix sent as part of hemicolectomy was normal in appearance but on sampling showed few well-defined glands. Initially confused for metastasis, the cells were noted to have bland nuclear features and surrounding these were rounded cells possibly representing endometrial stroma. Immunohistochemistry for estrogen receptor (ER) was performed and was positive in the glands and the stromal cells (Figure 1d and inset). Finally, a diagnosis of well-differentiated adenocarcinoma of the colon with appendiceal endometriosis was tendered.

Case 5 was a seven-day-old boy with meconium ileus, later proved to have cystic fibrosis. Appendicectomy was done to facilitate irrigation of colon. Also, the organ was bloated, thinned and filled with inspissated mucin. Slide showed evidence of focal mucosal mucinous hyperplasia in an overall attenuated epithelium, with thinned out muscularis mucosa and the lumen dilated, filled with inspissated mucin (Figure 1e). Overall, features favoured a mucocele, non-neoplastic type.

Case 6 was a 24-year-old woman with multiple small polyps in the duodenum, sigmoid colon and a pedunculated polyp coming out of the appendiceal orifice on endoscopy. It was sampled piecemeal and revealed to have classic features of hyperplastic polyp with focal ulceration and an overall increase in goblet cells (Figure 1f). No atypia or evidence of malignancy was noted.

Case 7 was a 27-year-old male patient, clinically resolved appendicitis. His appendix showed near-total obliteration of lumen with hypertrophied nerve bundles with wavy nuclei admixed with minimal fibroadipose tissue (Figure 1g). No tumor or evidence of ganglion cells/neuroendocrine cells was seen in the sections studied. It was labelled as a case of appendiceal neuroma/fibrous obliteration of the appendix.

Case 8 was a 27-year-old male who underwent emergency appendicectomy for acute appendicitis symptoms. The appendix was congested externally and had a yellowish, well-defined lesion about, 3 cm in diameter near the tip. Histopathological examination showed a classic (subserosal) lipoma (Figure 1h).

Case 9 was a suspected appendicular abscess with a bulky appendix on gross examination in a 56-year-old female. On cut section, a small tumor of 1 cm size near the base was seen which was uniloculated and had a glistening mucinous appearance. Microscopy revealed a localized tumor that shows a lining of low-grade dysplastic mucinous epithelium (Figure 1i). No high-grade nuclear features/cribriform glands, muscular invasion or mucin pools were noted. A final diagnosis of mucinous cystadenoma was given.

Case 10 was a 14-year-old boy who underwent an interval appendicectomy for resolved appendicitis and was found to have a 0.6-cm whitish tumor nodule at the tip of the appendix comprising nests and cords of monomorphic tumor cells with salt and pepper chromatin (Figure 1j and inset). Though the tumor appeared to invade the muscularis propria, the mitotic activity was negligible and the Ki-67 labelling index <2%. A diagnosis of well-differentiated neuroendocrine tumor (carcinoid) was rendered. The proximal margin being free, no additional therapy was deemed necessary.

Case 11 represents an adult male of 56 years with suspected appendicular malignancy and (multiple) liver secondaries and enlarged ileocolic nodes. Gross examination of the appendix revealed a 1.6-cm nodule at its tip, which on microscopy showed a transmural large cell lymphoma, immunohistochemically proven to be a diffuse large B-cell lymphoma, ulcerating the mucosal epithelium and extending into subserosal fat (Figure 1k and inset). Sampled local nodes showed partial effacement by lymphoma cells. The liver nodule excised also showed involvement by lymphoma cells. The pathologist called it a DLBCL of appendix with liver infiltration. The patient unfortunately succumbed after two cycles of chemotherapy.

Case 12 was a 53-year-old lady with extensive macroscopic peritoneal and colonic deposits (including in the cecum) and a left-sided tubo-ovarian mass. The tumor of the ovary was proven immunohistochemically to be serous papillary cystadenocarcinoma. Left uncommented on the surgical notes, the appendix (removed as part of segmental cecal resection) was firm and somewhat enlarged at the base while grossing. Microscopically, unequivocal tumor deposits in a desmoplastic stroma were noted (Figure 1l and inset).

The salient features of these 12 cases are summarized below in Table 1.

**Table 1:** Clinicopathological summary of representative rare and/or incidental appendiceal lesions from a single-center series. *M = Male; F = Female

Case no.	Age (in years)	Sex*	Gross (external) appearance	Microscopy	Co-existing disease	Region involved	Comment(s) on unique aspects
1	70	M	Unremarkable	Pinworm infection	Retroperitoneal epithelioid sarcoma	Lumen	Incidental, elderly, unrelated primary diagnosis
2	25	M	Unremarkable	Tuberculosis	Intra-abdominal tuberculous lymphadenopathy	Body	Rare site
3	49	F	Bulky	Xanthogranulomatous appendicitis	Nil	All	Rare diagnosis
4	81	F	Unremarkable	Appendiceal endometriosis	Adenocarcinoma of colon	Body	Incidental, postmenopausal endometriosis
5	7 days	M	Enlarged, thinned	Mucocele (non-neoplastic)	Cystic fibrosis	All	Rare diagnosis (for India)
6	24	F	Polyp (endoscopy)	Hyperplastic polyp	Nil	Base	Rare site
7	27	M	Unremarkable	Neuroma	Nil	All	Incidental, young age
8	27	M	Yellow lesion near tip	Subserosal lipoma	Nil	Tip	Rare site
9	56	F	Bulky	Mucinous cystadenoma	Nil	Body	Rare site
10	14	M	Unremarkable	Carcinoid	Nil	Tip	Incidental, young age
11	56	M	Nodule at tip	Appendiceal diffuse large B-cell lymphoma	Liver, intra-abdominal nodes involved by lymphoma	Tip	Rare site
12	53	F	Bulky tip, firm overall	Metastatic (ovarian carcinoma) deposits	Ovarian serous cystadenocarcinoma	Body	Incidental

Among the total 43 cases, 27 were truly incidental in that they were not identified on radiology or clinical evaluation. These “truly incidental” lesions comprised pinworm infection (11 cases), neuroma (nine cases), (small subcentimetric) carcinoids (three cases), two cases of granulomatous appendicitis (tuberculous origin) and one case each of endometriosis and metastatic deposits. The remaining 16 were less of a surprise since, the true mystery was the diagnosis and not the organ per se, i.e., the appendix had a morbidity explaining radiologically established lesion in them. On the grossing table, 25 cases (58%) had normal-appearing appendix externally. This is a potential harbinger for undersampling and the consequent underreporting of appendiceal pathology.

## Discussion

Historically considered a vestigial organ, of late, as is their wont, researchers have started unearthing reasons to resurrect this dead and buried organ called appendix at the high table. Basic histological knowledge reminds us of the dense immune tissue housed within the walls of this organ. However, credit to the workers who saw beyond the obvious and helped establish a potential causative role of the appendix in ulcerative colitis and its protective role in the recurrence of *Clostridium difficile* infection [1-3]. The appendix is now known to be a reservoir or “safe-house” for one’s indigenous intestinal microbiome. It helps repopulate the gut with familiar bacteria and maintain the critical protective biofilm of colon after an episode of diarrhoea or a bystander insult from antibiotic use [4,5]. Physiology apart, for pathologists the current fame of this humble organ is its role as the possible origin of pseudomyxoma peritonei [6].

Increasingly, with financial and manpower cost (for tissue processing) and tissue storage stress, there are thoughts gravitating towards the possibility of issuing reports, solely based on gross examination for those lesions that are obvious to the naked eye such as an appendiceal perforation, hydatidiform mole, fallopian tubes, lipoma and so on. As a response to that dangerous thought, we hoped to reinforce with our series that a myriad of potentially curable, troublesome and/or critical (such as for tumor staging) lesions are hidden (radiologically) or may manifest in even a normal-appearing appendix. Eventually, in our series, there were 27 and 25 examples of such lesions, respectively. These were among a total of 43 rare lesions reported over a decade at our center with a healthy average of around four non-routine diagnoses per year. In fact, analyzing retrospectively, it may not be an exaggeration to claim that these numbers would be higher if we had sampled each grossly normal-appearing appendix entirely with extensive, regular cross-sections. But then, a compromise based on practicality is essential and we feel confident enough with our limited, yet representative sampling strategy to recommend it to others as a bare minimum.

Worms are not unusual in the appendix. Pinworm infection is by far the commonest. It is common among young children, their primary caregivers as parents, the institutionalized with poor personal hygiene and in homosexuals [7]. Our patient, to the contrary, was an elderly man. He did, though, have a young grandchild at home and a big family. The other parasites reported in appendicectomy specimens include *Taenia* spp. (tapeworm), *Ascaris lumbricoides* (roundworm), *Trichuris trichiura* (whipworm), *Schistosoma haematobium*, *Schistosoma mansoni*, and the protozoan *Entamoeba histolytica* [8-11]. These have a fairly unique size and anatomy that hardly ever cause confusion with pinworms on histopathologic evaluation.

Granulomatous appendicitis is a rare entity with widely varied etiology. The broad classification would be infectious and non-infectious causes. Some of the various known infectious causes are Tuberculosis, *Yersinia* spp., Actinomycosis, Schistosomiasis and nematodes like pinworm. The famed non-neoplastic agents would include Crohn’s disease, sarcoidosis, foreign body such as vegetable matter, post-interval appendicectomy and the waste basket “idiopathic” category. While certain etiologies offer diagnostic clues, many require clinical correlation. For example, eosinophil-rich granulomas without a demonstrable parasite may still be considered as parasitic and the etiology narrowed down to local epidemiology [12,13]. Luckily, in one of our cases, the presence of prominent caseous necrosis and prominent Langhans cells led us to strongly suggest tuberculosis as the likely etiology though acid-fast bacilli were not demonstrable. Postoperatively, a CT scan showed intra-abdominal necrotic nodes. The patient responded to antituberculous therapy with improved general condition. One may note that in endemic zones it is more or less a thumb rule that even a non-caseating granuloma is to be considered as tuberculous until proven otherwise.

An interesting variant of granulomatous response is Xanthogranulomatous appendicitis. Only about 20 case reports exist in the literature to date on the entity. Overall, it may resemble neuroendocrine lesions in view of their yellow appearance and are said to be common in adults after interval appendicectomy though reports to the contrary exist [14,15]. No definitive etiology is known as yet. Proposed postulates include faulty lipid transport, immunologic disturbances as altered chemotaxis in inflammatory cells, *Proteus *sp*.* or *E. coli* infection, and lymphatic obstruction [15]. Our case was unique for its presentation as a tumorous mass to the operating surgeon.

Endometriosis in the appendix is, again, a known occurrence with incidence varying from 0.02 to 0.8% of all appendicectomies [16]. However, our patient presented a unique diagnostic dilemma. The clinical issue in question was the potential upstaging of the tumor from T2 to T4a because of the initial argument that the glandular structures were those of a metastatic deposit given the fact that she was operated for adenocarcinoma of the colon. However, a benign appearance resembling proliferative glands (positive for ER, on immunohistochemistry), ER-positive stromal cells and serosal location favoured endometriosis, though she was post-menopausal (80 years). We could find an occasional case report of post-menopausal endometriosis, including one involving the appendix. The putative mechanisms for postmenopausal endometriosis are hormone replacement therapy, the sufficiency of baseline estrogen levels, autocrine estrogenic self-sufficiency of endometriotic tissue and phytoestrogens in diet [17].

Polyps in the appendix conform to a similar classification as with other polyps seen in the gastrointestinal tract and are probably uncommon. No epidemiological data regarding the frequency of various polyps could be found in our literature search.

Appendiceal neuroma, previously known as fibrous obliteration of the appendix, is not a true neoplasm and represents hyperplasia of S-100 positive neural elements with a frequent but not compulsory obliteration of the lumen. Its prevalence is said to increase with age while our patient was a rather young adult. Its mimics would include tumors such as neurofibroma, schwannoma, neural predominant ganglioneuroma and gastrointestinal stromal tumor but these are extremely rare in the appendix [18].

Lipoma is a ubiquitous tumor. No surprise then, that it may affect the appendix or the periappendiceal tissue. Strangely though, case reports of “appendiceal” lipoma are few and far between and frequently in the context of complications such as torsion [19-21]. One may thereby infer that it may be perceived to be so common that is underreported or that it may genuinely be a rare entity, especially the subserosal type as was seen in our case.

Mucocele is by and large a diagnosis of gross evaluation and implies an appendix that is either entirely or focally distended with mucin. The etiology may be neoplastic or non-neoplastic. Non-neoplastic etiology includes obstruction of its lumen coupled with altered motility and proximal mucus pile-up from whatever cause [22]. Neoplastic origin may be due to the existence of mucosal hyperplasia/mucinous cystadenoma or mucinous cystadenocarcinoma [23]. Neoplastic mucoceles are more often localized and walled off uni- (occasionally multi-) loculated cystic lesions. The danger with these is the tendency to dissect through the layers of muscle leading to the feared pseudomyxoma peritonei [24]. In our series, the non-neoplastic spectrum was represented by a case of cystic fibrosis and the neoplastic by an incidental mucinous cystadenoma. Fortunately, pseudomyxoma had not developed in the patient. Cystic fibrosis, also known as mucoviscidosis, is another known unusual albeit logical cause of mucocele with the name itself being the giveaway [25]. An important differential in the western hemisphere, it is not common in India which makes our case unique.

Neuroendocrine neoplasms of the appendix account for up to four-fifth of all appendiceal neoplasms [26]. A majority are asymptomatic and in the tip of the appendix as was our case. The grade 1 tumors are the commonest. The bright yellow tinge of the tumor is not an infallible observation. Depending on the size, a definite tumor may be seen only on microscopy. Even then, liberal labelling may lead to overdiagnosis of neuroendocrine cell hyperplasias as carcinoids [18]. Criteria such as muscle involvement and gross nodule, and expansion of appendix reduce such misadventures. Overall, a grade 1 neuroendocrine tumor confined to the appendix tip with size of <2 cm and more definitively <1 cm with no nodal metastasis portends a good prognosis [27]. Our patient had these favourable factors and is doing well with no further treatment.

Primary appendiceal lymphomas are extremely rare being diagnosed in 0.015% of all appendicectomy specimens [28]. A majority are non-Hodgkin lymphomas of B-cell origin and present across a wide age range. Diffuse large B-cell lymphomas are considered most common but Burkitt lymphoma, follicular lymphoma, mantle cell lymphoma, small lymphocytic lymphoma, peripheral T-cell lymphoma and unclassifiable NHLs have all been reported [29]. Our patient had a nodule on the tip of the appendix which on microscopy seemed to have a transmural presence of the large lymphomatous cells raising the possibility of appendicular origin. However, the presence of intra-abdominal nodes, though unsampled, may argue against a primary appendiceal origin.

Non-contiguous metastasis to the appendix may not be difficult to diagnose but is important to note as clinical staging depends on it. We had a case of ovarian malignancy metastasis congruent with the literature cited by WHO that quotes common primary malignancies of the gastrointestinal and urogenital tract, breast, lung, gall bladder, thymoma and melanoma as culprits of appendiceal metastasis [30].

## Conclusions

To conclude, rare lesions of the appendix are not so rare that a full histopathological evaluation is to be deemed redundant. In our opinion, a meticulous microscopic evaluation of the appendix needs to be done each time it arrives in a histopathology laboratory, either singly or as part of more extensive surgeries. This is because the lesions detected may have therapeutic solutions or a future implication. Surgeons manning the smaller hospitals and nursing homes should sensitize suspected appendicitis patients regarding the need for this microscopic evaluation while the histopathologists and pathology assistants must be alert while sampling and examining an appendix.
